# microRNA-128-3p inhibits proliferation and accelerates apoptosis of gastric cancer cells via inhibition of TUFT1

**DOI:** 10.1186/s12957-023-02906-0

**Published:** 2023-02-17

**Authors:** Xiong Du, Yanxin Li, Bin Lian, Xiangli Yin

**Affiliations:** 1 Department of Pathology, Yanan University Affiliated Hospital, Yan’an, 716000 Shaanxi China; 2 Guangzhou Huayin Medical Laboratory Center. Ltd., Guangdong 510000 Guangzhou, China; 3 Department of Pathology, Xi’an International Medical Center Hospital, No.777, Xitai Road, High-Tech Zone, Xi’an, 710000 Shaanxi China

**Keywords:** Gastric cancer, MicroRNA-128-3p, Tuftelin1, Proliferation, Invasion, Epithelial-mesenchymal transition, Apoptosis

## Abstract

**Objective:**

Gastric cancer (GC) is a malignant tumor rooting in the gastric mucosal epithelium, ranking the first among various malignant tumors. Therefore, the influence of microRNA-128-3p (miR-128-3p) by regulation of Tuftelin1 (TUFT1) on GC cells was investigated.

**Methods:**

The expression levels of miR-128-3p and TUFT1 in GC tissues and cells were detected. The correlation between miR-128-3p expression and overall survival of GC patients was analyzed. Human GC cells MGC803 were transfected with miR-128-3p or TUFT1-related oligonucleotides to figure their roles in viability, apoptosis, invasion, as well as epithelial-mesenchymal transition (EMT). The relationship between miR-128-3p and TUFT1 was validated.

**Results:**

miR-128-3p expression was low and TUFT1 expression was high in GC tissues. miR-128-3p expression was positively correlated with the overall survival of patients with GC. miR-128-3p targeted TUFT1. Up-regulated miR-128-3p or suppressed TUFT1 repressed viability, invasion, and EMT, and accelerated apoptosis of GC cells. Overexpressed TUFT1 reduced miR-128-3p-mediated growth inhibition of GC cells.

**Conclusion:**

The study stresses that miR-128-3p can inhibit TUFT1 expression, thereby repressing GC cell activities.

## Introduction

Gastric cancer (GC) is the fifth most prevailing malignant tumor around the world and the third major reason of cancer-linked mortality [1]. GC, especially intestinal-type GC, progresses through developed changes from long-term gastritis to gastric atrophy, intestinal metaplasia, dysplasia, and invasive carcinoma [2]. Epidemiological changes are found in GC occurrence, with higher incidence rates in East Asia, Eastern Europe, and South America [3]. Helicobacter pylori takes on a decisive role in GC progression [4]. A social endoscopic screening system has promoted simple detection of early-stage GC and early GC (EGC) therapy has ameliorated endings, while the patient prognosis with advanced GC has not eased completely due to its high rate of metastasis and recurrence [5]. GC is known as poor prognosis and absence of effective treatment [6]. Therefore, it is essential to develop more feasible and effective therapeutic strategies for reducing damage caused by GC.

It is well demonstrated that microRNAs (miRNAs) have a clear influence on the management of all kinds of tumor development [7]. With reference to a recent publication, it is indicated that miR-128 is a key tumor suppressor, down-regulated in GC [8]. Guo et al. have described their findings in GC that miR-128 is involved in cisplatin resistance in the disease [9]. Meaningfully, Yu et al. have discussed the value of miR-128 for the diagnosis and prognosis of patients with GC [10]. Concerning to miR-128-3p, emerging studies have investigated its tumor-suppressing effects on cancer cellular progression [11, 12], but few was known about its role in regulating GC cell progression. Tuftelin1 (TUFT1) is an acidic protein component of developing and mineralizing tooth tissue that is related to oncogenesis of cancers. Actually, TUFT1 could induce invasion of triple negative breast cancer in a dose-dependent manner [13]. Mechanistically, in a clinical test, Zhou et al. have proved that overexpressed TUFT1 is associated with lymph node metastasis and advanced tumor stage of patients with pancreatic cancer [14]. On a bioinformatics website, we predicted the targeting relation between miR-128-3p and TUFT1, thus it was inferred that miR-128-3p may regulate the progression of GC cells through TUFT1. Therefore, the destination of this study was for the investigation of the effect of miR-128-3p on GC cells by modulation of TUFT1.

## Materials and methods

### Ethical approval

The study was permitted by the Institutional Review Board of Xi’an International Medical Center Hospital and followed the tenets of the Declaration of Helsinki. All patients in this experiment signed informed consent.

### Cases of specimens

A total of 103 patients with primary GC diagnosed and treated in Xi’an International Medical Center Hospital were selected. Patients were included if they followed that (1) primary GC was confirmed by cytology and histopathology without radiotherapy or chemotherapy; (2) age > 18 years, complete clinical data including gender, age, Tumor (T) stage, N (node) stage, clinical grade, and pathological stage; (3) no other primary tumors; no serious liver, lung, kidney, or other chronic diseases; (4) all patients underwent radical gastrectomy for GC and the corresponding GC and adjacent normal tissue specimens were obtained.

In situ cancer specimens and adjacent normal tissue specimens were excised from the 103 patients. The excised tissues were rapidly fixed with 10% formaldehyde and then paraffin sectioned for immunohistochemical analysis or rapidly frozen in liquid nitrogen and stored at − 80℃ for later use.

### Immunohistochemistry

All specimens were fixed with 10% formaldehyde, embedded in paraffin, and sectioned in sections of 4 μm. After de-waxing and hydration, TUFT1 expression in tissue specimens of patients was detected via immunohistochemistry. Endogenous peroxidase in the tissue section was blocked with 1% hydrogen peroxide for 10 min and then further blocked with 1% goat serum in PBS for 30 min. The tissue sections were treated with the primary antibody TUFT1 (1:50, Abcam) at 4℃ overnight, added with the secondary antibody for 10 min, and stained with diaminobenzidine (DAB). Microscopically, TUFT1 positive expression was observed in the cytoplasm, showing a brown-yellow color.

### Cell culture

GC cell lines (MGC803, BGC823, and SGC-7901) and gastric epithelial cells (GES-1) were provided by ATCC (VA, USA) and cultured in Roswell Park Memorial Institute (RPMI) 1640 medium containing 10% fetal bovine serum, 100 U/mL penicillin, and 100 μg/mL streptomycin at 37℃. When the cell confluence reached 70–80%, the cells were passaged.

### Cell grouping

Cells were divided into groups: miR-Ctrl group (transfected with miR-128-3p mimic negative control [NC]), miR-128-3p group (transfected with miR-128-3p mimic), si-Ctrl (transfected with TUFT1 siRNA NC vector), si-TUFT1 (transfected with TUFT1 siRNA vector), miR-128-3p + overexpressed (oe)-TUFT1 group (transfected with miR-128-3p mimic + pcDNA-TUFT1 vector), and miR-128-3p + oe-Ctrl group (transfected with miR-128-3p mimic + pcDNA 3.1 empty vector).

MGC803 cells were seeded into six-well plates containing RPMI 1640 medium (2 × 10^5^ cells/well) and cultured at 37℃. When the cell confluence was about 90%, the cells were transfected in line with the instructions of Lipofectamine™ 2000 Transfection Reagent (Thermo Fisher Scientific, USA), and three replicates were set for each treatment. All oligonucleotides or plasmids were purchased from GenePharma (Shanghai, China).

### MTT assay

The cells were cultured with 20 μL 5 g/L MTT solution (Gibco, CA, USA) at the 24^th^, 48^th^, and 72^nd^ h of culture. Each well was joined with 150 μL dimethyl sulfoxide and then shaken on the micro-plate reader to fully dissolve the crystals. The optical density (OD_490 nm_) value was measured. Five replicates were set for each group.

### Flow cytometry

The transfected cells were harvested and rinsed with PBS three times. With the removal of the supernatant, the cells were resuspended in a binding buffer, added with 5 μL annexin V-fluorescein isothiocyanate, and 10 μL propidium iodide. The apoptosis was detected by a flow cytometer.

### Transwell assay

After detachment by trypsin, the cells were re-suspended. The cell suspension (200 μL) was seeded into the Transwell chamber (Corning, NY, USA) covered with matrix gel, and cultured on the lower chamber containing complete medium for 24 h. The cells were fixed with 4% paraformaldehyde, stained with 0.1% crystal violet solution, and photographed under the microscope to count the number of membrane penetrating cells.

### RT-qPCR

Total RNA was extracted from tissues and cells via Trizol reagent (Invitrogen, CA, USA). After the concentration and purity of RNA samples were determined by spectrophotometer, RNA was reversed-transcribed into cDNA in line with the instructions of PrimeScript RT reagent kit (Takara, Dalian, China) and Mir-X miR First-Strand Synthesis kit (Takara). PCR primers were synthesized by ComWin Biotech (Beijing, China) (Table 1). Glyceraldehyde-3-phosphate dehydrogenase (GAPDH) was the loading control for mRNA (U6 for miRNA). The data were analyzed via using 2^−ΔΔCt^ method.

**Table 1:** Primers for used genes in PCR method

Genes	Sequences
miR-128-3p	Forward: 5'-TCACAGTGAACCGGTCTCTTT-3'
	Reverse: Universal primer
U6	Forward: 5'-CGCTTCGGCAGCACATATAC-3'
	Reverse: 5'-AAATATGGAACGCTTCACGA-3'
TUFT1	Forward: 5'-AAAGGACGCCACCATCCAG-3'
	Reverse: 5'-GTGCTGAAGTTGCCATGACTG-3'
GAPDH	Forward: 5'-AACGGGAAGCTCACTGGCATG-3'
	Reverse: 5'-TCCACCACTGTTGCTGTAG-3'
Bcl-2	Forward: 5'-TGTGGATGACTGACTACCTGAACC-3'
	Reverse: 5'-CAGCCAGGAGAAATCAAACAGAGG-3'
Bax	Forward: 5'-GGGGACGAACTGGACAGTAA-3'
	Reverse: 5'-CAGTTGAAGTTGCCGTCAGA-3'
E-cadherin	Forward: 5'-CAGCATCACTGGCCAAGGAGCTGA-3'
	Reverse: 5'-GACCACACTGATGACTCCTGTGTTCC-3'
Vimentin	Forward: 5'-CCGACACTCCT ACAAGATTTAGA-3'
	Reverse: 5'-CAAAGATTTATTGAAGCAGAACC-3'
N-Cadherin	Forward: 5'-TTTGATGGAGGTCTCCTAACACC-3'
	Reverse: 5'-ACGTTTAACACGTTGGAAATGTG-3'

miR-128-3p, microRNA-128-3p; TUFT1, Tuftelin1; GAPDH, Glyceraldehyde-3-phosphate dehydrogenase

### Western blot analysis

The proteins in tissues and cells were extracted and protein concentration was measured by bicinchoninic acid method. The extracted protein was mixed with loading buffer at a ratio of 2:1, boiled for denaturation, implemented with sodium dodecyl sulfate polyacrylamide gel electrophoresis, and transferred onto polyvinylidene fluoride membrane. Then the membrane was blocked and incubated at 4℃ overnight with primary antibodies TUFT1 (1:1000) and GAPDH (1:1000, Abcam, Cambridge, MA). The membrane was incubated with horseradish peroxidase-labeled secondary antibody (1:5000, Abcam) for 1 h and developed by enhanced chemiluminescence chemical method. The relative expression of target protein was calculated by using the gray value analysis software.

### Dual luciferase reporter gene assay

Bioinformatics software RNA22 (https://cm.jefferson.edu/rna22/Precomputed/) was used for predicting the binding sites between miR-128-3p and TUFT1. Sequences of TUFT1 3′UTR promoter regions containing miR-128-3p binding sites were synthesized to construct TUFT1 3′UTR wild-type (WT) plasmid (TUFT1-WT). The binding sites were mutated to construct TUFT1 3′UTR mutant type (MUT) plasmid (TUFT1-MUT). When the MGC803 cell confluence was about 70%, miR-128-3p and miR-Ctrl were mixed with TUFT1-WT and TUFT1-MUT plasmids, respectively, and then co-transfected into MGC803 cells. The cells were lysed after 48-h transfection, and the luciferase activity was detected via a luciferase assay kit (Promega, Madison, WI, USA).

### Statistical analysis

SPSS 21.0 statistical software (IBM Corp. Armonk, NY, USA) was used for data analysis. The measurement data were expressed in the form of mean ± standard deviation. Statistical analysis between the two groups was performed using either paired *t*-test or independent samples *t*-test. One-way analysis of variance (ANOVA) was applied for comparisons among multiple groups, and after ANOVA analysis, Tukey’s post hoc test was utilized for pairwise comparison. Pearson test was implemented for correlation analysis. Kaplan–Meier (K-M) survival curve and Log-rank test were utilized for determining the total survival of patients. Enumeration data were presented with number and analyzed by Fisher’s exact test or Chi-square test. Predictors were kept if they were significant at a *P* value of 0.05 or smaller.

## Results

### miR-128-3p expression is low and TUFT1 expression is high in GC tissues

**Fig. 1:**
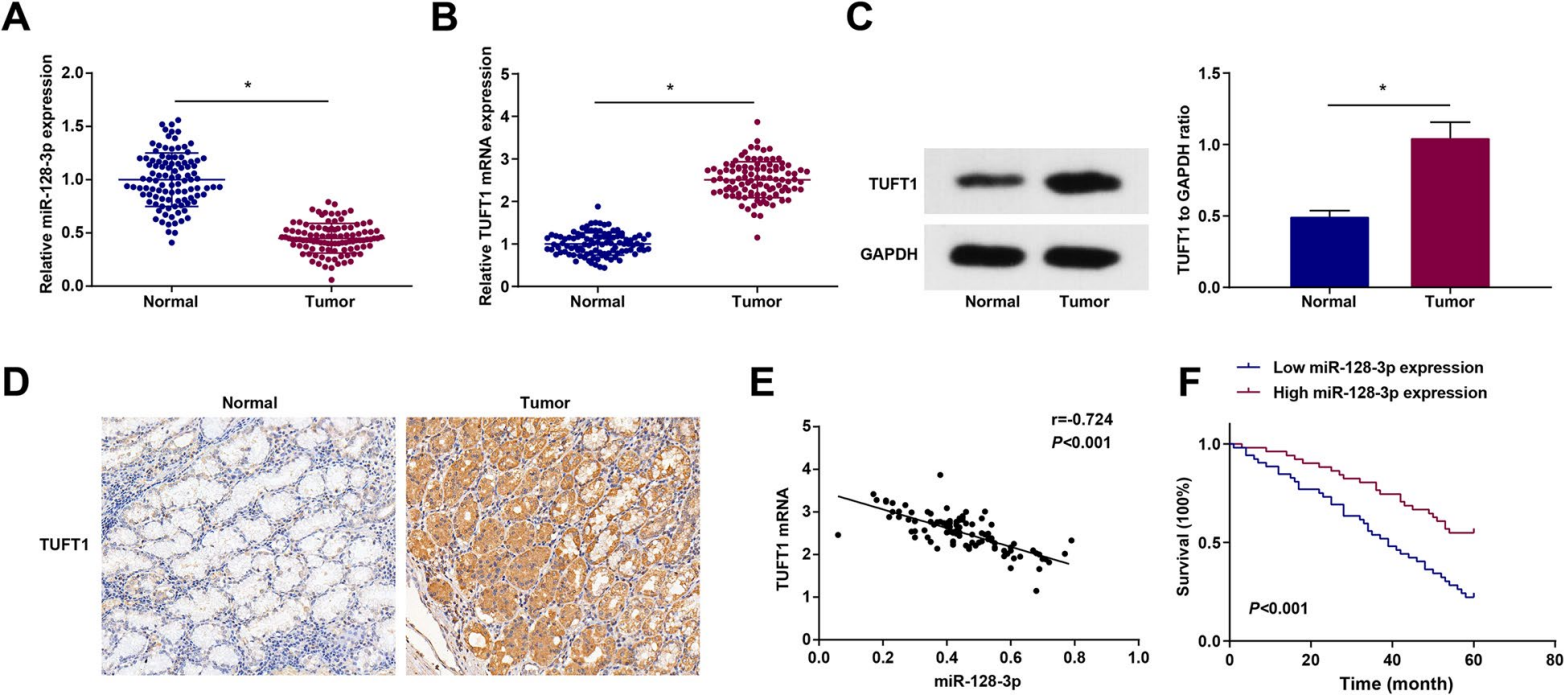
miR-128-3p expression is low and TUFT1 expression is high in GC tissues. Low expression of miR-128-3p is associated with poor prognosis in patients with GC. **A** The expression of miR-128-3p in GC tissues and adjacent normal tissues detected via RT-qPCR. **B** The expression of TUFT1 in GC tissues and adjacent normal tissues detected via RT-qPCR. **C** The protein expression of TUFT1 in GC tissues and adjacent normal tissues detected via western blot analysis. **D** TUFT1 expression in GC tissues and adjacent normal tissues detected via immunohistochemistry. **E** The correlation between the expression of miR-128-3p and TUFT1 mRNA in GC patients analyzed via Pearson test. **F** Kaplan–Meier analyzed the effect of miR-128-3p on the survival and prognosis of GC patients. *P < 0.05

In GC tissues and adjacent normal tissues, miR-128-3p levels were detected by RT-qPCR, while TUFT1 levels by RT-qPCR, Western blot analysis, and immunohistochemistry (Fig. 1A–D). The results showed that TUFT1 expression was higher, and miR-128-3p was lower in GC tissues than in normal tissues.

Pearson’s test was further applied to analyze the correlation between miR-128-3p and TUFT1 mRNA expression in GC tissues and the results found that (Fig. 1E) there was a negative correlation between miR-128-3p and TUFT1 mRNA expression in GC tissues.

### Low expression of miR-128-3p is associated with poor prognosis in patients with GC

To evaluate the effect of miR-128-3p on the prognosis of patients with GC, 103 patients were divided into high expression group and low expression group based on the median of miR-128-3p expression. Table 2 showed that low miR-128-3p expression levels were correlated with tumor size, tissue differentiation, TNM stage, depth of invasion, and distant metastasis. To further study the clinical value of miR-128-3p in GC, Kaplan–Meier method was applied to analyze the survival of 103 patients with GC, and the results showed that the overall survival of patients with high miR-128-3p was higher than that of patients with low miR-128-3p (*P* < 0.05, Fig. 1F).

**Table 2:** Correlation between miR-128-3p expression and clinicopathological characteristics of gastric cancer

Clinicopathological characteristics	*n*	miR-128-3p		*P*
	103	Low expression (*n* = 52)	High expression (*n* = 51)	
Age				0.429
≤ 59 years	59	32	27	
> 59 years	44	20	24	
Gender				0.158
Male	40	24	16	
Female	63	28	35	
Tumor size				0.028
≤ 4.8 cm	59	24	35	
> 4.8 cm	44	28	16	
Tissue differentiation				0.009
Well	12	3	9	
Moderate	46	19	27	
Poor	45	30	15	
TNM stage				< 0.001
I + II	48	14	34	
III + IV	55	38	17	
Distant metastasis				0.005
M0	42	14	28	
M1	61	38	23	
Infiltrating depth				0.001
T1 + T2	42	13	29	
T3 + T4	61	39	22	

### Screening of cell lines

**Fig. 2:**
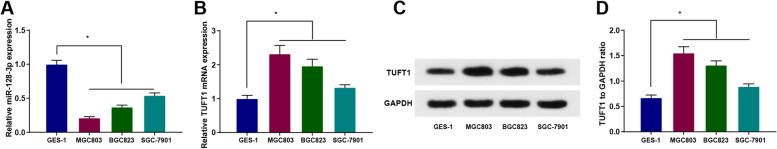
Screening of cell lines. **A-B** miR-128-3p and TUFT1 expression in GC cell lines and gastric mucosal epithelial cells detected by RT-qPCR. **C-D** TUFT1 expression in GC cell lines and gastric mucosal epithelial cells detected by Western blot. *P < 0.05

To study the effects of miR-128-3p and TUFT1 on GC in vitro, their expression was assessed in GC cell lines (MGC803, BGC823, and SGC-7901) and GES-1 by RT-qPCR and Western blot. As expected, miR-128-3p was lowly expressed while TUFT1 was highly expressed in GC cell lines, especially in MGC803 cells (Fig. 2A–D), so subsequent experiments were performed with MGC803 cells.

### Targeting relation between miR-128-3p and TUFT1

**Fig. 3:**
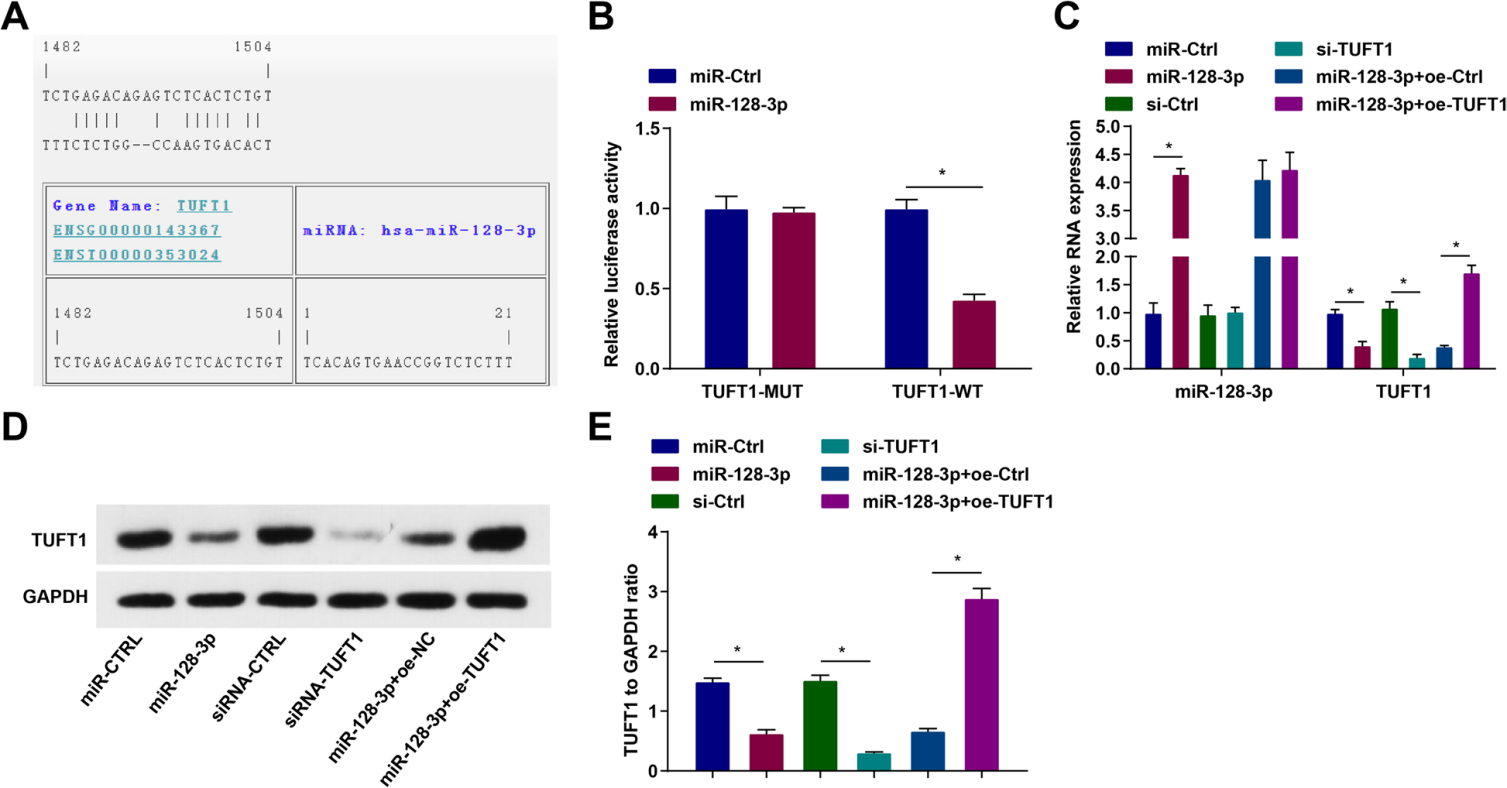
Targeting relation between miR-128-3p and TUFT1. **A** The binding sites of TUFT1 and miR-128-3p predicted by Jefferson. **B** The targeting relationship between TUFT1 and miR-128-3p verified by dual luciferase reporter gene activity test. **C** miR-128-3p and TUFT1 levels in MGC803 cells detected by RT-qPCR. **D-E** TUFT1 protein expression in MGC803 cells detected by Western blot. *P < 0.05

To explore the targeting relationship between miR-128-3p and TUFT1, bioinformatics software RNA22 (https://cm.jefferson.edu/rna22/Precomputed/) was utilized for analysis of the targeting sites between miR-128-3p and TUFT1 (Fig. 3A). Also, the experimental results of luciferase activity assay showed that (Fig. 3B) miR-128-3p had no obvious effect on the luciferase activity of TUFT1-MUT plasmid, but reduced that of TUFT1-WT plasmid.

Next, RT-qPCR and Western blot were performed to detect miR-128-3p and TUFT1 levels in MGC803 cells after transfection (Fig. 3C–E). It was found that transfection with miR-128-3p mimic elevated miR-128-3p whilst reduced TUFT1 expression in MGC803 cells; however, transfection with TUFT1 siRNA reduced TUFT1 expression in MGC803 cells; moreover, transfection with TUFT1 overexpression vector could reverse the role of miR-128-3p mimic on regulating TUFT1 expression.

### Elevated miR-128-3p inhibits the biological activities of GC cells

**Fig. 4:**
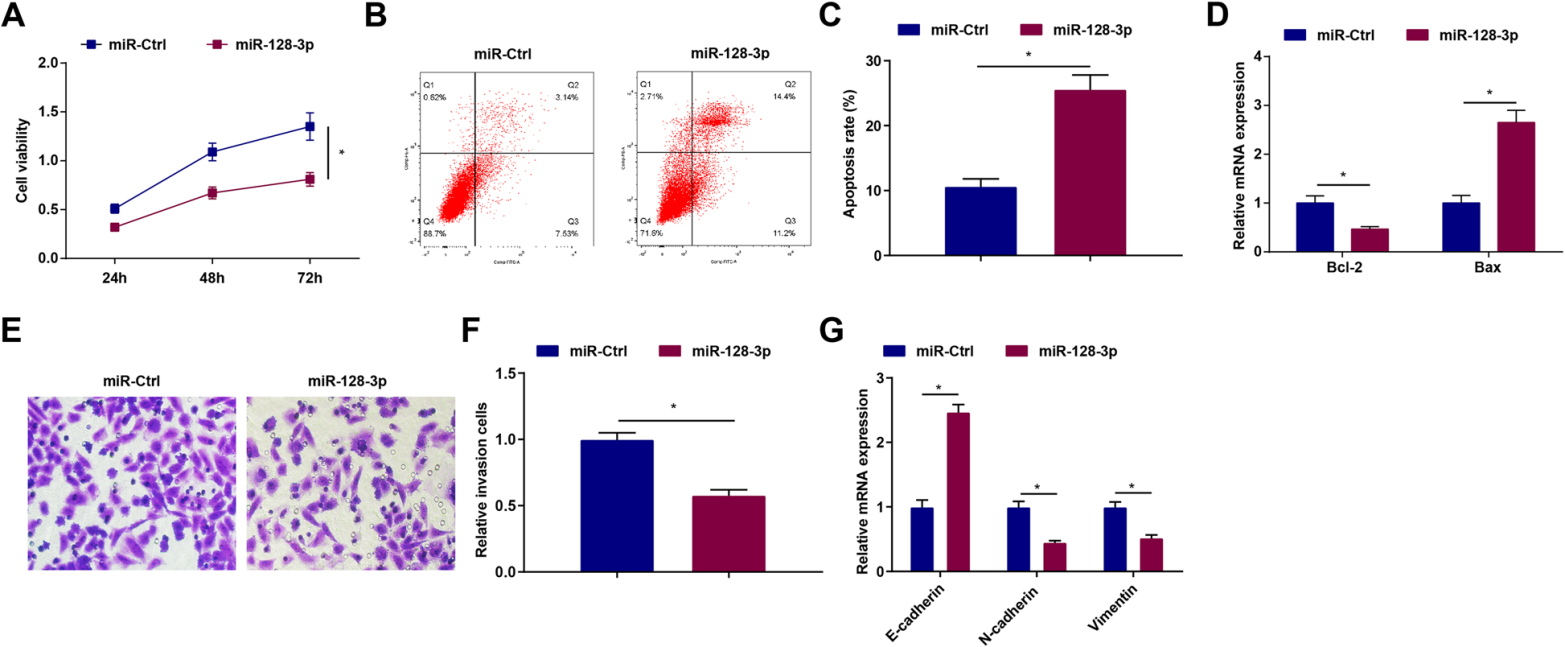
Elevated miR-128-3p impedes the biological functions of GC cells. **A** The cell viability of MGC803 cells after transfection with elevated miR-128-3p detected via MTT assay. **B-C** The apoptosis of MGC803 cells after transfection with elevated miR-128-3p detected via flow cytometry. **D** Bcl-2 and Bax mRNA expression in MGC803 cells after transfection with elevated miR-128-3p detected via RT-qPCR. **E–F** The invasion ability of MGC803 cells after transfection with elevated miR-128-3p detected via Transwell assay. **G** The mRNA expression levels of E-cadherin, N-cadherin, and Vimentin after transfection with elevated miR-128-3p tested by RT-qPCR. *P < 0.05

miR-128-3p is under-expressed in colorectal cancer [15]. To explore the effect of miR-128-3p on GC, miR-128-3p mimic was transfected into MGC803 cells, which up-regulated miR-128-3p expression as suggested above. Through MTT assay, it was obviously detected that in MGC803 cells containing up-regulated miR-128-3p, cell viability was suppressed (Fig. 4A). Then, flow cytometry, along with RT-qPCR, was utilized to measure cell apoptosis, and the results demonstrated that overexpressing miR-128-3p elevated apoptosis rate and Bax mRNA expression while reduced Bcl-2 mRNA expression (Fig. 4B–D). Meanwhile, cell invasion was tested by Transwell assay with the outcome suggesting that miR-128-3p-overexpressing MGC803 cells had decreased ability of invasion (Fig. 4E, F). Furthermore, the evaluation of the expression levels of the EMT-related factors unveiled that miR-128-3p-overexpressing MGC803 cells exhibited elevated E-cadherin level and reduced N-cadherin and Vimentin levels (Fig. 4G).

### Knockdown of TUFT1 depresses the biological activities of GC cells

**Fig. 5:**
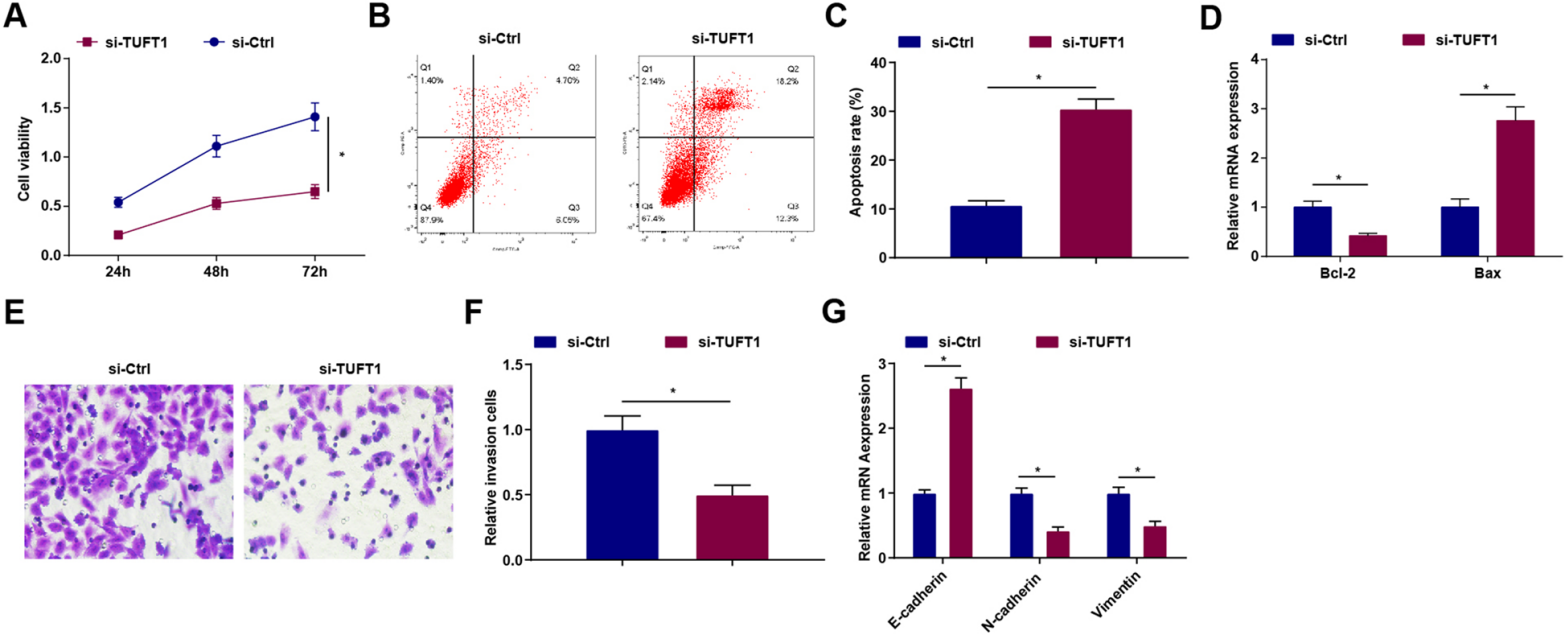
Knockdown of TUFT1 depresses the biological functions of GC cells. **A** The cell viability of MGC803 cells after transfection with silenced TUFT1 detected via MTT assay. **B-C** The apoptosis of MGC803 cells after transfection with silenced TUFT1 detected via flow cytometry. **D** Bcl-2 and Bax mRNA expression in MGC803 cells after transfection with silenced TUFT1 detected via RT-qPCR. **E–F** The invasion ability of MGC803 cells after transfection with silenced TUFT1 detected via Transwell assay. **G** The mRNA expression levels of E-cadherin, N-cadherin, and Vimentin after transfection with silenced TUFT1 tested by RT-qPCR.*P < 0.05

TUFT1 could inhibit the growth of pancreatic cancer cells [14], but there are few studies on the effect of TUFT1 on GC. Thus, we explored the role of TUFT1 in GC cell development. Using siRNA transfection, we successfully knocked down TUFT1 expression in MGC803 cells as detected above. In response to TUFT1 expression reduction, it was subsequently examined that the viability, invasion, and EMT were retarded while apoptosis rate was elevated, Bcl-2 mRNA expression was reduced and Bax mRNA expression was induced in MGC803 cells (Fig. 5A–G).

### Up-regulation of TUFT1 reverses the effect of miR-128-3p overexpression on GC cells

**Fig. 6:**
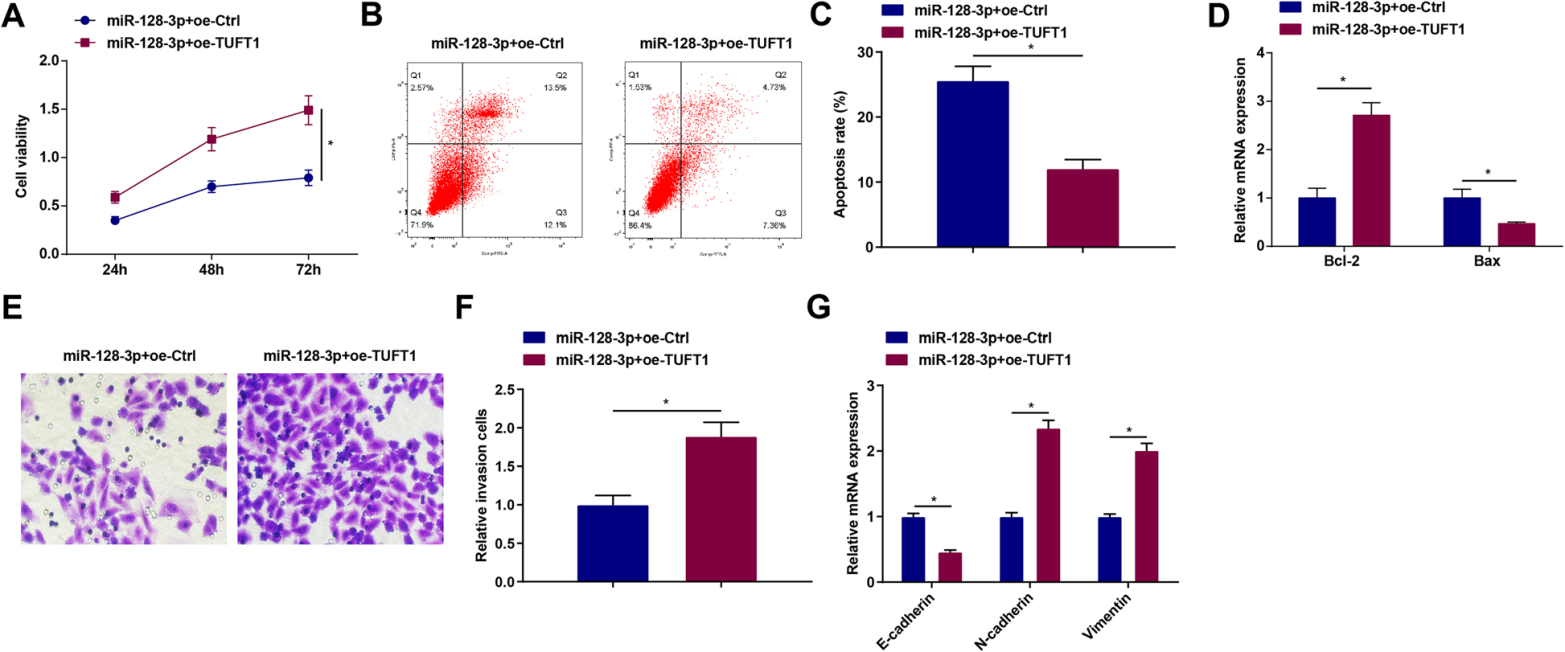
Up-regulation of TUFT1 reverses the effect of miR-128-3p overexpression on GC cells. **A** The cell viability of MGC803 cells detected via MTT assay. **B-C** The apoptosis of MGC803 cells detected via flow cytometry. **D** Bcl-2 and Bax mRNA expression in MGC803 cells detected via RT-qPCR. **E–F** The invasion ability of MGC803 cells detected via Transwell assay. **G** The mRNA expression levels of E-cadherin, N-cadherin, and Vimentin tested by RT-qPCR. *P < 0.05

Finally, to explore the effect of miR-128-3p on GC cells through targeted regulation of TUFT1, miR-128-3p mimic and TUFT1 overexpression vector were transfected into MGC803 cells. The results showed that TUFT1 overexpression vector reversed miR-128-3p mimic-induced impacts on viability, EMT, apoptosis rate, Bcl-2 and Bax mRNA expression, and invasion ability of MGC803 cells (Fig. 6A–G).

## Discussion

GC is a familiar malignancy of the digestive system and exhibits a serious burden to human health [16]. In the course of GC, we found that miR-128-3p could suppress the malignant activities of GC cells through regulation of its target TUFT1.

The major result of the study was that miR-128-3p was down-regulated in cancer tissue samples of patients with GC and was negatively associated with tumor size, tissue differentiation, TNM stage, depth of invasion, and distant metastasis. Also, the results further confirmed the prognostic value of miR-128-3p in patients with GC. It has been significantly elaborated that lowly expressed miR-128-3p in lung cancer tissue samples of patients is correlated with TNM stage and tumor size [17]. Kang et al. have defined that there is a negative correlation between low expression of miR-128-3p and poor prognosis, tumor stage, and differentiation of patients with hepatocellular carcinoma [18]. As to the biological regulation of miR-128-3p for GC cells, our observational outcomes indicated that miR-128-3p overexpression suppressed viability, invasion, and EMT, whereas induced apoptosis of MGC803 cells. In a cell-based model, it is interestingly investigated that miR-128-3p could reduce viability, induce apoptosis, and increase the accumulation of intracellular oxaliplatin, thereby delaying the development of colorectal cancer [15]. In addition to that, it is evidenced that miR-128-3p exerts an anti-tumorigenesis effect in the case of esophageal squamous-cell cancer, as reflected by inhibiting metastatic activity of cancer cells and delaying the process of epithelial-mesenchymal transition [19]. On the other hand, Wang et al., have presented the data that miR-128-3p inhibition accelerates proliferation and limits apoptosis of cervical cancer cells [20]. Also, in non-small cell lung cancer cells introduced with miR-128-3p inhibitor, it could be seen that viability and invasion are phenotypically stimulated [21]. EMT is a vital process that happens in the process of tumor metastasis, which influences diverse malignancies. It is reported that exosomal miR-128-3p modulates EMT by directly repressing its downstream target gene FOXO4 [22]. Also, Zhang et al. have stated that miR-128-3p participates in the EMT process of lung cancer cells [23].

An obvious result of our study was that miR-128-3p targeted TUFT1 and negatively regulated TUFT1 expression. To our best knowledge, the relation between the two was reported before, which is the novelty of the present study. With regard to TUFT1, our research found that it was overexpressed in GC clinical samples and experimental cell lines. In the meantime, the present study captured an outcome that TUFT1 silencing was inhibitory for the growth of MGC803 cells, but TUFT1 overexpression could counteract the inhibitory effects of miR-128-3p on MGC803 cells. Liu et al. have observed that TUFT1 is overexpressed in breast cancer [24] and further explained that the metastasis and stem cell-like trait of TUFT1-silenced triple negative breast cancer cells are inhibited [25]. Moreover, a publication concerning to pancreatic cancer has illustrated that TUFT1 expression is induced in the disease progression and TUFT1 has the capacity to accelerate growth, metastasis, and epithelial-mesenchymal transition of cancer cells [26]. In the development of cervical cancer, up-regulating TUFT1 is effective in increasing proliferation and reducing apoptosis of drug-resistant tumor cells [27]. Significantly, Dou et al. have tested the involvement of overexpressed TUFT1 in the progression of hepatocellular carcinoma, and the activated TUFT1/AKT pathway could enhance cellular growth and metastasis [28]. In addition to the above cancers, TUFT1 has been found to be overexpressed in osteosarcoma, and TUFT1 induction serves aggressively for the viability, migration, and invasion of osteosarcoma cells [29]. As for its roles in EMT, a publication has demonstrated that depletion of endogenous TUFT1 impacts the expression levels of EMT-associated proteins (E-cadherin and Vimentin) in pancreatic cancer [14]. Also, Lin et al. have mentioned that TUFT1 overexpression stimulates the EMT progression of renal cell carcinoma cells while downregulation of TUFT1 retards such process [30].

## Conclusion

**Fig. 7:**
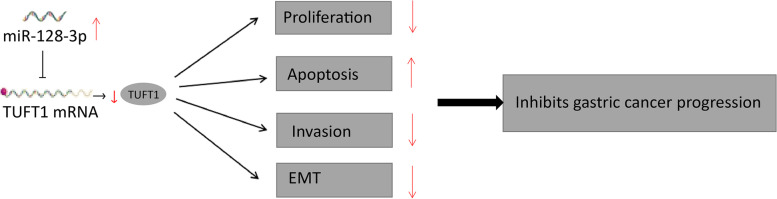
Mechanism diagram: overexpression of miR-128-3p impedes cell proliferation, invasion, and EMT and promotes cell apoptosis by inhibiting TUFT1

All in all, the study stresses that miR-128-3p can inhibit TUFT1 expression, thereby suppressing viability and invasion and facilitating apoptosis of GC cells (Fig. 7). This study may have promising effects on attenuating GC and may be of significance from the aspect of cell biological processes. Despite several of researches, the mechanisms and treatments for GC have not been completely understood. Therefore, further studies are needed to explore effective therapies for GC.

## Data Availability

The data that support the findings of this study are available from the corresponding author upon reasonable request.
